# Annual and Seasonal Variability of Trichloromethane in Drinking Water of Kunshan City 2016–2022 and Associated Health Risks

**DOI:** 10.3390/toxics12120865

**Published:** 2024-11-28

**Authors:** Xiaojun Liang, Guohua Qian, Yihan Wang, Mengyao Chen, Yang Liu, Ping Zhao, Junling Li, Yuan Wang, Yuyan Liu

**Affiliations:** 1 Center for Disease Control and Prevention of Kunshan, Kunshan 215300, China; lxjbj1984@163.com (X.L.); qianguohua@163.com (G.Q.); chenmy199591@163.com (M.C.); liuyangcl@126.com (Y.L.); 13776335097@163.com (P.Z.); 2 School of Public Health, Suzhou Medical College, Soochow University, Suzhou 215127, China; yihanwangnnn@163.com (Y.W.); tljunling@163.com (J.L.)

**Keywords:** trichlormethanes, drinking water, Kunshan, DBPs, health risk

## Abstract

This study aimed to evaluate the annual pollution characteristics of trichloromethane (TCM) in Kunshan City’s tap water from 2016 to 2022. This research analyzed 566 tap water samples from centralized water supply units, utilizing the GB 5749-2006 Sanitary Standard for Drinking Water as the evaluation benchmark. Data analysis employed non-parametric tests and Spearman’s correlation analysis using Excel 2017 and SPSS 26.0. The results indicated a 100% compliance rate with the TCM limit (0.06 mg/L), with median annual concentrations ranging from 0.1 to 6.4 μg/L. Significant inter-annual variations were observed (H = 222.5, *p* < 0.01), with the lowest levels in 2019 and the highest in 2020. Quarterly analysis revealed significant seasonal differences (H = 94.0, *p* < 0.01), peaking in the third quarter (8.0 μg/L) and bottoming in the first quarter (3.5 μg/L). TCM concentrations showed significant correlations with annual and quarterly trends, turbidity, and chlorides (|rs| > 0.3, *p* < 0.01) but not with pH (rs = −0.0025, *p* = 0.55). While Kunshan City’s drinking water demonstrates satisfactory TCM levels, an increasing annual trend and higher concentrations in the latter half of the year warrant continued monitoring and investigation. In this study, we assessed the health risks for households in Kunshan, China, due to trichloromethane (TCM) in drinking water. The overall carcinogenic risk from multiple exposure pathways was slightly above the ideal level, while the non-carcinogenic risk was within an acceptable range.

## 1. Introduction

Currently widely used chlorination and chloramination disinfection technologies produce large amounts of disinfection by-products (DBPs) that pose health risks to humans [1]. Among these, trihalomethanes (THMs) were first discovered in 1974 and are the first chemical category of DBPs [2].

The formation of trihalomethanes (THMs) during water chlorination is a complex process influenced by numerous factors, primarily the characteristics of raw water, water quality parameters, and the type, dose, and contact time of the disinfectant used. Natural organic matter (NOM), especially in surface water, is the main precursor for THM formation [3]. NOM consists of hydrophobic components, such as humic and fulvic acids, as well as hydrophilic components, including proteins and carbohydrates [4,5]. The concentration of dissolved organic carbon (DOC) in raw water is strongly positively correlated with THM levels [6]; the higher the DOC concentration, the greater the amount of THMs formed [7]. Water quality parameters such as temperature and turbidity also significantly influence THM production [8,9,10]. Higher temperatures and turbid waters, which contain higher microbial and organic loads, further increase THM formation. The chlorine dose and contact time are also crucial; longer exposure to chlorine and higher doses lead to increased THM levels due to more extensive reactions with NOM [9].

The choice of disinfectant plays a key role in THM formation [11,12,13]. Chlorine is the most commonly used disinfectant and readily reacts with NOM to form THMs. However, other disinfectants such as chloramine and chlorine dioxide can reduce THM formation [14], but they may produce different by-products. Understanding the interaction between water quality parameters and disinfectant types is essential for minimizing THM formation and improving water quality management.

THMs are commonly found in tap water and wastewater treatment processes, including trichloromethane (TCM), bromodichloromethane (BDCM), dibromochloromethane (DBCM), and tribromomethane (TBM), with TCM being the most abundant and common [15]. Numerous domestic and international epidemiological studies and toxicological experiments have shown that THMs have carcinogenic, mutagenic, and teratogenic effects [16,17,18,19]. Among them, TCM, DBCM, and TBM have been classified as B2 carcinogens by the U.S. Environmental Protection Agency, while BDCM is classified as a C carcinogen. It has been confirmed that THMs are associated with digestive system cancers such as bladder cancer, colon cancer, and rectal cancer [20,21,22]. China’s “Hygienic Standard for Drinking Water” GB5749-2022 stipulates [23] that the concentration of trichloromethane in tap water shall not exceed 0.06 milligrams per liter (0.06 mg/L). The United States Environmental Protection Agency requires [24] that the sum of the concentrations of trichloromethane, bromodichloromethane, dibromochloromethane, and tribromomethane, collectively known as total trihalomethanes (THMs), must not exceed 0.08 milligrams per liter (0.08 mg/L) in drinking water. Table 1 presents the approximate concentrations of trihalomethanes in various countries and regions. As a result, some countries have failed to keep THM concentrations in drinking water within regulatory limits, which could pose health risks to consumers. Therefore, regular monitoring of DBPs in chlorinated water is necessary.

This study analyzes water quality monitoring data from Kunshan City from 2016 to 2022 to provide reliable data support for TCM risk management by the Kunshan Water Supply Group Co., Ltd. (Shaoqing East Road, Kunshan City, China). It aims to reasonably assess the health risks of local residents’ exposure to TCM, which is of great significance for ensuring the safety of drinking water.

## 2. Materials and Methods

### 2.1. Water Plant Status and Sample Collection

There are two centralized sources of drinking water in Kunshan: P puppet Lake and the Yangtze River. The P puppet Lake water source is located in the Bachi Town area and is classified as a lake-type source. The Yangtze River water source is located in Hupu, Changshu, and is classified as a river-type source. Both water sources meet the national surface water Class II quality standard. Water from the Yangtze River is transported to Kunshan through two DN2200 raw water pipelines and mixed with the water from P puppet Lake before being sent to various water treatment plants for processing. Currently, Kunshan’s daily water supply capacity is 1.5 million cubic meters, provided by the Jinghe Water Plant, the Third Water Plant, and the Fourth Water Plant. The Jinghe Water Plant has an outflow pipeline of approximately 15 km, primarily supplying water to the old city area and northern rural areas. The Third Water Plant has an outflow pipeline of approximately 30 km, primarily supplying water to Bachi Town, parts of the Development Zone, and eastern rural areas. The Fourth Water Plant has an outflow pipeline of about 4 km, primarily supplying water to parts of the Development Zone and southern rural areas.

The Kunshan Water Treatment Plants (WTPs) use a conventional water treatment process consisting of pre-chlorination, coagulation, sedimentation, sand filtration, activated carbon filtration, and post-chlorination. Gaseous chlorine is directly added to the pumped raw water at the WTP inlet (i.e., the intake pump station) for pre-chlorination. The final disinfection at the WTP is achieved using chloramine. The chlorine dosage varies depending on the WTP and the season. However, the average free chlorine residual after chlorination at the WTP is relatively constant at 0.98 mg/L.

**Figure 1:**
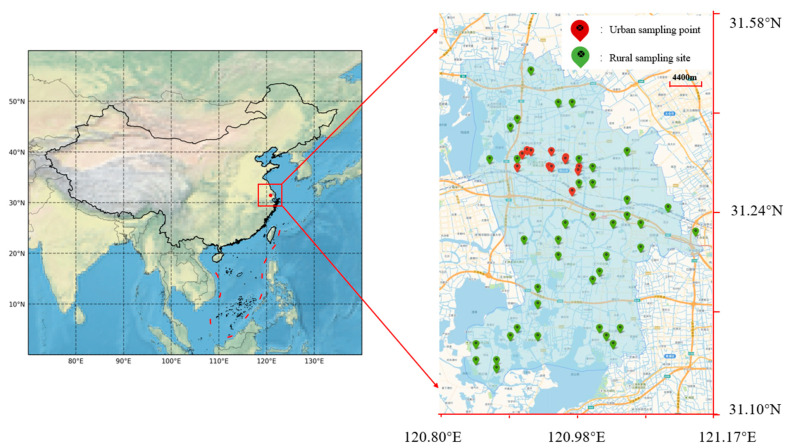
Administrative divisions of Kunshan and sampling point locations.

A total of 40 rural drinking water monitoring points were established in 10 towns of Kunshan City (Figure 1), including 2 water treatment plant outlets and 38 end-of-pipe locations. In the urban area, 12 drinking water monitoring points were set up, comprising 1 water treatment plant outlet, 5 end-of-pipe locations, and 6 secondary water supply points. Water samples were collected quarterly from these locations.

For routine monthly monitoring, 3 water treatment plant outlets and 13 end-of-pipe locations were designated. Rural drinking water monitoring was conducted twice a year, during the dry season (February) and wet season (August). Urban drinking water monitoring was carried out quarterly (February, May, August, and November), while routine monitoring was performed monthly.

The total annual monitoring frequency satisfied the requirements for rural, urban, and routine monitoring simultaneously. Monitoring results from centralized water supply units collected by the Kunshan Center for Disease Control and Prevention between 2016 and 2022 were compiled. Out of 569 water samples monitored, 566 were included in the analysis after excluding some samples with missing data.

### 2.2. Methods

#### 2.2.1. Detection Method

Prior to sample collection, the water taps were disinfected and then allowed to run for approximately 15 min. Samples were collected directly into sterilized sampling bottles, taking care to avoid contamination of the bottle’s mouth by fingers or other objects. For physicochemical parameter samples, sampling equipment, containers, and stoppers were rinsed 2 to 3 times with the sample water before collection.

Samples were tested according to GB/T 5749-2022 “Standard Examination Methods for Drinking Water”, with the trichloromethane concentration limit set at 0.06 mg/L [20]. Parameters tested included trichloromethane, pH, chloride, and turbidity.

Trichloromethane was detected using the headspace capillary gas chromatography method [47]. Water samples were placed in sealed headspace vials and allowed to reach equilibrium at a specific temperature for a set time. Trichloromethane volatilized into the headspace, achieving a dynamic equilibrium between gas and liquid phases. The concentration of trichloromethane in the gas phase is proportional to its concentration in the liquid phase. The gas phase was then analyzed using a gas chromatograph with an ECD detector. By measuring the trichloromethane concentration in the gas phase, the concentrations of trichloromethane and carbon tetrachloride in the water sample could be calculated.

#### 2.2.2. Analysis Method

Data were processed and analyzed using Microsoft Office Excel 2017 and Statistical Product and Service Solutions software (version 26.0, SPSS). As the concentrations of the analyzed compounds did not follow a normal distribution, non-parametric tests were used to evaluate annual and seasonal variations in trichloromethane concentrations. Spearman correlation tests were employed to analyze correlations between variables. The significance level was set at α = 0.05 (two-tailed), with *p* < 0.05 considered statistically significant.

## 3. Results and Discussion

### 3.1. Annual Variation Characteristics of Trichloromethane

**Figure 2:**
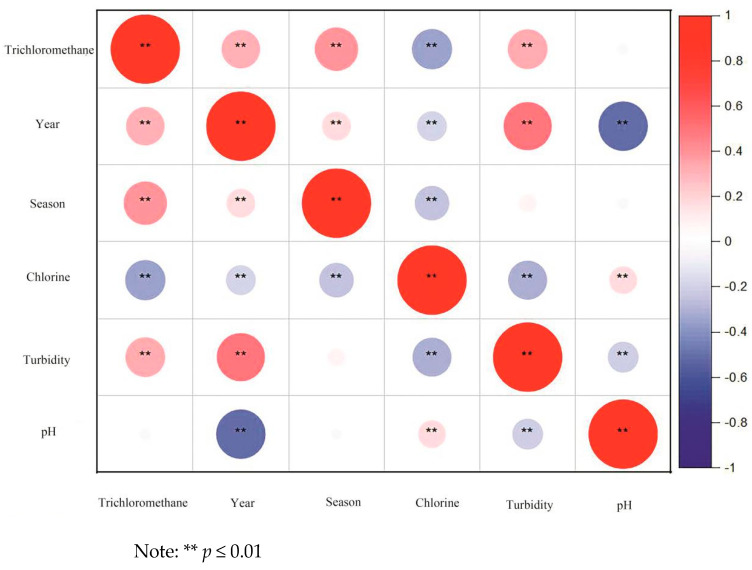
Spearman correlation of chloroform with year, season, chloride, turbidity, and pH.

The trichloromethane component in drinking water was analyzed and summarized based on the sampling year. The median concentrations of trichloromethane for each year were 2.3 μg/L, 2.0 μg/L, 0.3 μg/L, 0.1 μg/L, 6.4 μg/L, 4.4 μg/L, and 5.0 μg/L, respectively. The maximum values for all years were significantly lower than 60 μg/L. The median concentration was the lowest in 2019 and the highest in 2020, showing an overall trend of initial decrease, followed by an increase, and then a decrease again. The differences between years were statistically significant (Table 2). A correlation analysis was performed with year as the independent variable and trichloromethane as the dependent variable. The Spearman correlation coefficient (*r_s_*) between the two was 0.308, with *p* < 0.01, indicating a weak positive correlation (Figure 2).

**Figure 3:**
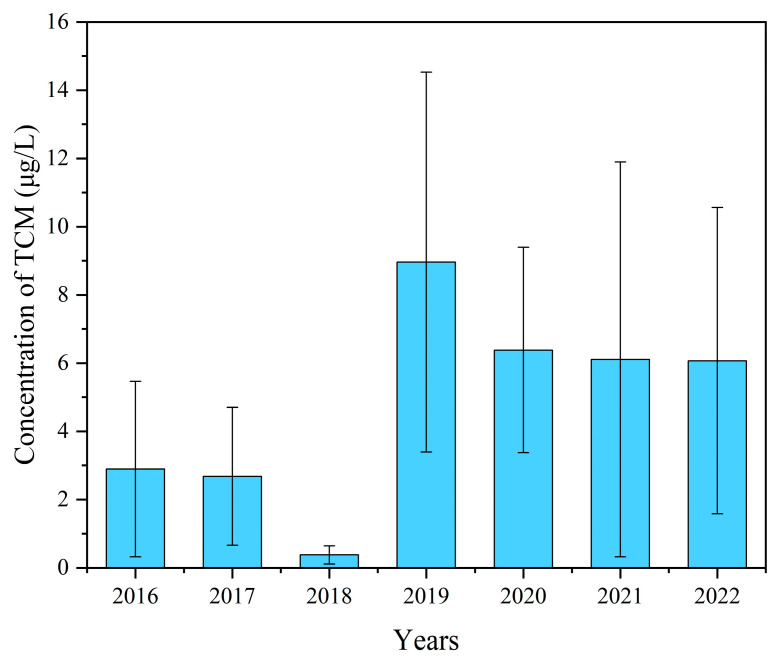
Trichloromethane concentration in terminal water of Kunshan City from 2016 to 2022.

The water quality monitoring results from 2016 to 2022 indicate that the trichloromethane content in the terminal water of Kunshan City remained at persistently low levels, complying with the relevant national water quality standards. Over the years, the trichloromethane concentration in drinking water exhibited a general upward trend. In 2018, the median trichloromethane content reached its lowest point but peaked in the following year. In the absence of changes in sampling locations, this phenomenon could be attributed to differences in sample size and adjustments in sampling times. The 2018 data set comprised only 44 samples, less than one-third of the 134 samples collected in 2019, which might account for the apparent shift in trichloromethane levels between adjacent years. However, the sample sizes in 2016, 2017, 2018, and 2020 were relatively comparable, yet the trichloromethane concentrations differed significantly between 2017 and 2018, as well as between 2018 and 2020 (Figure 3). This suggests that sample size alone cannot explain the inter-annual variations in trichloromethane levels. Upon examining the data collection schedules, there is reason to suspect that the observed discrepancies might stem from temporal differences in sampling periods within each year.

### 3.2. Seasonal Variation Characteristics of Trichloromethane

The trichloromethane component in drinking water was analyzed and summarized based on the sampling quarter. The median concentrations of trichloromethane for each quarter were 3.5 μg/L, 3.8 μg/L, 8.0 μg/L, and 5.9 μg/L, respectively. The concentration of trichloromethane was highest in the third quarter, showing an overall trend of initial increase followed by a decrease. The differences between quarters were statistically significant (Table 3). Using the quarter as the independent variable and trichloromethane as the dependent variable, a correlation analysis was performed. The Spearman correlation coefficient (*r_s_*) between the two was 0.378, with *p* < 0.01, indicating a weak positive correlation (Figure 2).

Trichloromethane concentrations exhibited notable quarterly variations, steadily increasing from the first to the third quarter, peaking, and then declining in the fourth quarter. This pattern is likely influenced by seasonal temperature fluctuations. On one hand, elevated temperatures may accelerate the chemical reactions responsible for trichloromethane formation. On the other hand, trichloromethane’s low boiling point of 61.2 °C facilitates its volatilization and escape from water at higher temperatures. Zhang et al. [48] proposed a kinetic model describing the temperature effects on DBP formation, indicating that higher temperatures significantly increase DBP formation rates and maximum attainable concentrations, with the latter exhibiting an approximately exponential growth pattern as temperature rises. Seasonal changes also impact the levels of organic precursor substances present in the source water. During warmer seasons, the abundant proliferation of aquatic plants and microorganisms contributes to increased total organic carbon content.

When compared with the results of a study on drinking water TCM concentrations in Suzhou (2018–2022), we found similarities in seasonal variation. The study in Suzhou reported TCM concentrations ranging from ND to 57.00 μg/L, with the median concentration at 8.00 μg/L. The study also found that TCM concentrations during the wet season (flood season) were significantly higher than during the dry season (*p* < 0.01), with concentrations in the wet season ranging from 10.5 to 17.45 μg/L, while the dry season concentrations were between 4.2 and 8.36 μg/L [49]. These findings align with our observations in Kunshan, suggesting that seasonal variations, precipitation, and fluctuations in the levels of organic precursors in water play a crucial role in determining TCM concentrations. In particular, during the wet season, increased rainfall leads to higher concentrations of organic precursor substances in the water, which in turn promotes the formation of TCM during the disinfection process.

The vast majority of organic matter in water, such as humic substances and fulvic acids, are the primary precursors for the formation of organic halogenated compounds, including trihalomethanes [50,51,52]. Concurrently, the third quarter coincides with Kunshan City’s wet season, during which heightened precipitation carries more substances into nearby water bodies, leading to elevated levels of organic precursors and, consequently, increased DBP formation during the disinfection process.

### 3.3. Influence of Water Components on Trichloromethane Content

#### 3.3.1. Chloride

Analysis of 566 samples showed a median chloride concentration of 28.6 mg/L, with an interquartile range of 9.9. All samples were within the permitted range of health standards (≤250 mg/L). A correlation analysis with chloride as the independent variable and trichloromethane as the dependent variable yielded a Spearman correlation coefficient (*r_s_*) of −0.317, with *p* < 0.01, indicating a weak negative correlation (Table 4, Figure 2).

Regarding the mechanism by which chlorides promote THM formation, chloride concentrations typically increase with increasing chlorine dosages, indirectly reflecting the magnitude of chlorine addition. As the chlorine dosage increases, the formation potential of THMs also rises [53]. Furthermore, the chlorine content influences the distribution of different THM species. When the chlorine-to-carbon ratio is less than 0.5, the reaction initially produces lower-oxidation-state chlorinated compounds. At a given concentration of organic matter, the formation of trihalomethanes exhibits a positive correlation with the chlorine dosage within a certain range. Studies suggest that the presence of chlorides facilitates the formation of phenolic THMs, and this increase in THMs occurs without an accompanying increase in chlorine consumption, as the chlorine demand is largely unaffected by the presence of chlorides [54]. As chloride concentrations rise, the percentage of THMs relative to the total initial carbon increases. However, even in the presence of chloride ions and free chlorine reactions, less than 1% of the initial carbon ultimately converts to THMs within 72 h, reflecting a relatively slow reaction rate. In the present study, the chloride levels exhibited a weak negative correlation with trichloromethane concentrations in the water, deviating from the generally observed trend.

The observed weak negative correlation between chloride and trichloromethane concentrations may be due to the water treatment process, including the dosage of chlorine and specific treatment methods such as coagulation, flocculation, and filtration, all of which can affect the chloride concentration in water. This in turn could influence the formation of trichloromethane. Under different water treatment conditions, the impact of chloride on trichloromethane formation may not be as pronounced as initially expected, particularly when variations exist in disinfectant dosage, type, and contact time.

#### 3.3.2. Turbidity

Analysis of 566 samples showed a median turbidity of 0.196 NTU, with an interquartile range of 0.0985. All samples were within the permitted range of health standards (≤1 NTU). A correlation analysis with turbidity as the independent variable and trichloromethane as the dependent variable yielded a Spearman correlation coefficient (*r_s_*) of 0.351, with *p* < 0.01, indicating a weak positive correlation (Table 4).

Turbidity is an indicator of water quality. Generally, higher turbidity suggests a higher content of DBP precursors, leading to increased disinfection by-products [55]. Tsitsifli and Kanakoudis [56] reported a stronger correlation between turbidity and THMs (*r* = 0.553) for two treatment plants using surface water sources. Our analysis, however, showed only a weak positive correlation between turbidity and chloroform concentration, with a lower correlation strength than Tsitsifli and Kanakoudis’s findings [56]. This discrepancy might be due to the following: (1) our data not following a normal distribution, necessitating rank correlation analysis using Spearman’s correlation coefficient, which may yield different values. (2) Our focus is solely on chloroform’s correlation with turbidity, while other THMs (dichlorobromomethane, chlorodibromomethane, and bromoform) might have more significant correlations with turbidity. (3) The impact of the water treatment process on turbidity and its relationship with trihalomethane formation is also worth considering. The treatment process can reduce turbidity by removing suspended solids and organic matter that may serve as precursors for DBPs (disinfection by-products). Therefore, any positive correlation between turbidity and chloroform concentration could be influenced by the efficiency of treatment and operational conditions during sample collection. If the treatment process effectively reduces turbidity, the relationship between turbidity and trihalomethane formation may be weaker than what would be expected based solely on raw water quality.

#### 3.3.3. pH

Analysis of 566 samples showed a median pH of 7.67, with an interquartile range of 0.25. All samples were within the permitted range of health standards (6.5–8.5). A correlation analysis with pH as the independent variable and trichloromethane as the dependent variable yielded a Spearman correlation coefficient (*r_s_*) of −0.025, with *p* = 0.55, indicating no significant correlation (Table 4, Figure 2).

The pH level can influence the primary reaction pathways and the speciation of various disinfectants, thereby affecting the formation and distribution of THM species. Under acidic conditions, the presence of hydrogen ions in water reduces the reactivity of disinfectants with THM precursors, resulting in lower THM formation rates. Conversely, in alkaline conditions, the increased pH facilitates the deprotonation of acidic functional groups in THM precursors, enhancing the reactivity of potential substitution sites and accelerating halogen substitution reactions, thus promoting THM formation [57]. However, the data analysis in this study revealed no apparent correlation between trichloromethane levels in Kunshan’s drinking water and the pH of the water bodies. This lack of correlation could be attributed to the narrow pH range of 6.5–8.5 specified for the actual water samples, which may not encompass a sufficiently wide range to observe significant effects. Previous literature suggests that at highly alkaline pH levels, chlorination can significantly increase THM formation, with pH 11 leading to higher trihalomethane formation potential compared to pH 8 [58]. Hua and Reckhow reported that the level of THMs at pH 10 is three times higher than at pH 5 [59]. The relatively narrow pH range of 6.5–8.5 observed in the samples may not fully capture the pH effects on THM formation, which could explain the lack of a strong correlation.

### 3.4. Health Risk Assessment of Chloroform in Drinking Wate

In recent years, there has been extensive research on the human health risks associated with THMs both domestically and internationally. There is increasing focus on measuring the concentrations of toxic substances and assessing the cancer risks related to THM exposure in public water supplies. Chloroform (TCM), as one of the most common DBPs, has been a key focus of research. This study conducted a health risk assessment following the guidelines of the United States Environmental Protection Agency (USEPA). Our assessment focused on both cancer and non-cancer risks associated with TCM exposure in drinking water. Three exposure routes were considered: oral ingestion, dermal absorption, and inhalation. Population exposure parameter values were sourced from the “Handbook of Chinese Population Exposure Factors” [60]. Chemical parameters for DBP health risks were obtained from the Integrated Risk Information System (IRIS) and the Risk Assessment Information System (RAIS). The health risk assessment for chloroform should include several steps [61]: Hazard Identification, Exposure Assessment, Dose–Response Relationship, and Risk Characterization.

#### 3.4.1. Hazard Identification

Hazard identification is the primary step in health risk assessment. With few exceptions, chloroform shows no mutagenic or genotoxic effects in various systems and endpoints, both in vivo and in vitro. All THMs possess carcinogenic properties [62]. Chloroform is a regulated THM that, based on data primarily obtained from animal studies, has been shown to cause liver and kidney tumors in mice and kidney tumors in rats [63]. Many studies indicate that chloroform is not genotoxic, and tumor induction occurs only at doses that produce significant cytotoxicity, cell death, and regenerative proliferation [63,64,65]. IRIS concluded from three different types of quantitative assessments for chloroform [66]: Under high exposure conditions, chloroform is likely to be carcinogenic to humans through all routes of exposure, leading to cytotoxicity and regenerative hyperplasia in susceptible tissues. However, under exposure conditions that do not induce cytotoxicity and cell regeneration, chloroform is unlikely to be carcinogenic to humans through any route of exposure.

Chloroform is the primary component among THMs in drinking water. THMs have a short elimination half-life in the body, less than one hour. After ingestion or inhalation of THMs, they are typically well-absorbed, metabolized, and rapidly eliminated [65]. THMs are distributed throughout the body, with the highest concentrations found in fat, blood, the liver, kidneys, lungs, and the nervous system. A considerable number of tissues can metabolize THMs, with the liver being the most important metabolic site. Metabolism primarily occurs through phase I and II metabolism via the cytochrome P450 mixed enzyme system in the liver, with metabolic products mainly being carbon dioxide and carbon monoxide [64]. Therefore, the liver is the primary target organ for human THM exposure.

#### 3.4.2. Risk Characterization

The main exposure routes for chloroform are oral ingestion, dermal absorption, and inhalation. Showering, bathing, and drinking water are considered the primary pathways for dermal, inhalation, and ingestion exposure [67]. Unit cancer risk is calculated through the Chronic Daily Intake (CDI) and the corresponding slope factor (SF), while hazard quotients and indices are assessed by calculating reference doses (RfDs) and reference concentrations. The CDI for multiple pathway assessment is calculated as follows [68]:

(1) $$CDIoral=\frac{C_{w-i}\times IRw\times EF\times ED}{BW\times AT}$$

(2) $$CDIdermal=\frac{C_{w-i}\times SA\times Kp\times ET\times EF\times ED}{BW\times AT}$$

(3) $$CDIinhalation=\frac{Cair\times IRa\times ET\times EF\times ED}{BW\times AT}$$

where C is the concentration in water samples (mg/L), IRw is the ingestion rate of drinking water (L/d), EF is the exposure frequency (d/year), ED is the exposure duration (years), BW is body weight (kg), AT is the expected lifespan (d), SA is the skin surface area of the human body (cm^2^), Kp is the skin permeability coefficient (cm/h), ET is the exposure time (h/event), Cair is the chloroform concentration in the breathing air zone (mg/m^3^), and IRa is the inhalation rate (m^3^/d) [69,70]. The TCM concentration in water samples is taken as 0.00426 mg/L, and AT (life expectancy) is 70 years. Other population exposure and chemical parameter values used for health risk calculations are listed in Table 5.

The unit cancer risk for each chlorinated by-product species through multiple exposure routes is calculated as follows:

(4) $$Rm=\sum_{i}SFm\times CDIm$$

(5) $$Hazardous\;Index=\frac{CDIingestion}{RfD}+\frac{CDIdermal}{GIABS*RfD}+\frac{CDIinhalation}{RfC}$$

where Rm is the unit risk estimate for compound m (dimensionless probability), i is the exposure route, SF (mg/kg/d)^−1^ is the slope factor value, CDI is the long-term daily intake, and the reference dose for Rm is 1 × 10^–6^ [70]. SFO = is the oral cancer slope factor of the DBPs (mg/kg/day)^−1^, (3.10 × 10^−2^ (mg/kg-day)^−1^), and RfD is the oral cancer reference dose of the DBPs (mg/kg/day), 1.00 × 10^−2^ mg/kg-day. GIABS is the gastrointestinal absorption factor (1). IUR and RfC are the inhalation unit risk (μg/m^3^)^−1^ and reference concentration (mg/m^3^) [68,71].

#### 3.4.3. Multiple Pathway Assessment of TCM Cancer Risk

**Figure 4:**
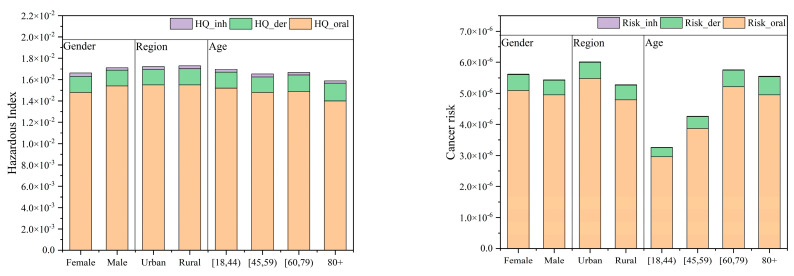
The cumulative cancer risk and hazardous index in different subgroups.

Table 6 and Figure 4 present the cumulative carcinogenic and non-carcinogenic risks through three exposure pathways for various subgroups. The calculations considered non-carcinogenic risk (using ED as AT) and carcinogenic risk (using 70 years as AT). The total risk values for TCM through multiple pathways ranged from 3.26 × 10^−6^ to 6.01 × 10^−6^, exceeding the generally accepted level of 1 × 10^−6^ but remaining within the upper limit of 1 × 10^−4^. The urban group exhibited the highest carcinogenic risk (6.01 × 10^−6^), while the [18, 44) age group showed the lowest (3.26 × 10^−6^).

The elevated carcinogenic risk in urban areas may be attributed to several factors: Water sources in urban regions are potentially subject to more industrial and domestic pollution, leading to higher organic content in raw water and increased trichloromethane formation. Additionally, urban water supply systems are typically more complex with longer pipeline networks and higher disinfection intensities, resulting in extended water retention times and higher residual chlorine concentrations, which may contribute to increased trichloromethane production.

Different exposure pathways contribute varying degrees to carcinogenic risk. Ingestion is the primary contributor, followed by dermal contact, with inhalation having a relatively minor impact. The dominance of the ingestion pathway can be explained by the daily necessity of drinking water, which directly enters the body through the digestive system, allowing for more immediate contact with bodily tissues. Moreover, trichloromethane absorbed through the digestive system may be more readily assimilated and metabolized by the body, potentially resulting in higher absorption efficiency. The risk through oral exposure is approximately 10 times that of dermal exposure and over 300 times that of inhalation.

Population differences are notable, with significant variations among age groups, primarily due to different exposure durations (EDs). Carcinogenic risk generally increases with age, possibly due to cumulative exposure time or age-related physiological changes. The slight decrease in risk for the 80+ age group may be attributed to various factors, such as reduced exposure time from bathing or showering due to lifestyle changes, decreased metabolic rates leading to slower accumulation of harmful substances, and drier, thicker skin potentially reducing absorption rates of certain chemicals.

However, actual health risk exposure may be lower than the results suggest. Heating causes significant volatilization of THMs, and considering that Chinese residents often heat tap water before consumption, related studies and surveys in similar regions [63] indicate that 56% of people drink heated tap water, with 78% of those continuing to heat for one minute after boiling. This process can reduce trichloromethane in water by 68%, resulting in lower actual ingestion-related carcinogenic risk.

In terms of non-carcinogenic risk, the HI values range from 1.59 × 10^−2^ to 1.72 × 10^−2^, with the lowest values observed in the 80+ age group. All groups have HI values below 1, indicating that the non-carcinogenic risk is within an acceptable range. Among the different exposure routes, ingestion contributes the most to non-carcinogenic risk, followed by dermal contact, while inhalation contributes relatively little. The risk via the oral route is approximately nine times greater than that via the dermal route and over 400 times greater than that via inhalation. Regarding population differences, the variation in non-carcinogenic risk among different groups is relatively small. However, there is a downward trend, possibly because younger individuals may more frequently engage in activities that increase exposure, such as long showers, swimming, or water sports. To ensure ongoing disinfection and prevent secondary contamination of the water supply system, a certain level of free residual chlorine is maintained in the water distribution network. Consequently, elevated water temperatures during showers may increase the reaction rate between residual natural organic matter and chlorine, leading to a higher formation of TCM in shower water. Additionally, trichloromethane is volatile and can easily be inhaled. Although non-carcinogenic HI values decrease with age, this does not imply that the overall health risks faced by the elderly are lower; carcinogenic risk may still accumulate with age.

In comparison, the Li study, based on TCM concentrations of 8.8–13.5 µg/L in tap water from Suzhou, reported a carcinogenic risk index of 1.5 × 10^−5^ and a non-carcinogenic HI index of 2.9 × 10^−2^. Although the risk values differ slightly, the results are very similar in magnitude and trend, suggesting that our findings align closely with those in the existing literature [71].

These findings suggest that while non-carcinogenic risks remain within acceptable limits, carcinogenic risks are slightly above ideal levels. Consequently, it is crucial to continue public education efforts to raise awareness about drinking water safety and promote the use of household water treatment devices. Regular monitoring of water quality and health impacts should be maintained to allow timely adjustments to risk management strategies.

## 4. Conclusions

In summary, from 2016 to 2022, the concentration of trichloromethane in Kunshan City’s water supply system remained below the national standard limit (0.06 mg/L), with annual median concentrations ranging from 0.1 to 6.4 μg/L. The overall trend initially declined, then rose, and subsequently declined again, exhibiting significant inter-annual variations and pronounced seasonal fluctuations. The third quarter exhibited the highest median concentration (8.0 μg/L), while the first quarter had the lowest (3.5 μg/L), with trichloromethane levels positively correlated with both year and season. Furthermore, a weak negative correlation was observed with the chloride ions in the water and a weak positive correlation with turbidity, whereas no significant correlation existed with pH. In this study, we assessed the health risks for households in Kunshan, China, due to trichloromethane (TCM) in drinking water. The overall carcinogenic risk from multiple exposure pathways was slightly above the ideal level, while the non-carcinogenic risk was within an acceptable range.

Although the overall trichloromethane levels in Kunshan’s drinking water were relatively low and compliant with standards, the upward annual trend and higher concentrations in the latter half of the year warrant continued monitoring and an in-depth investigation into the underlying causes. Relevant regulatory authorities should strengthen the supervision of water sources, production processes, and distribution networks to ensure the safety of public drinking water supplies. In conclusion, this study provides a comprehensive evaluation of the trichloromethane pollution situation in Kunshan City’s drinking water, revealing that the current water quality is under control; however, potential health risks should be acknowledged, and necessary preventive measures should be implemented.

## Figures and Tables

**Table 1:** Concentration of THMs in different countries.

Area	Location	Total Concentration (µg/L)	Reference
China	six cities	Max: 92.8	[25]
	Taiwan	ND-133.2	[26]
	Pearl River Delta	0.7–62.7	[27]
	31 cities	0.8–107	[28]
	35 cities	1.5–94.9	[29]
	Shenzhen	1.3–74.8	[30]
	Shandong	2.1–105	[31]
	Rural eastern regions	3.3–69.3	[32]
	Zhejiang	9.1–40.9	[33]
North America	Amherst	0–190 (TCM)	[34]
	USA	30–44	[35]
	North Carolina	39–82	[36]
	USA	Max: 164	[37]
	Canada	0.3–342	[38]
	Canada	38.1–111	[39]
	Canada	Max: 106	[40]
EU	Sweden	0.2–25	[41]
	Italy	168	[42]
	Spain	58–91	[43]
	UK	27.6–50.9	[44]
		BDL-83	[45]
	France	Max: 88.6	[46]

**Table 2:** Annual trichloromethane concentrations and rank-sum test results.

Item	Year	Sample Size	Range (μg/L)	Median (μg/L)	Interquartile Range	*W*	*p*	Mean Rank	H (K)
TCM	2016	44	0.02~12.98	2.31	1.94	0.69	<0.01	154	223
	2017	44	0.44~8.83	2.01	2.23	0.85	<0.01	153	
	2018	44	0.02~1.11	0.31	0.30	0.92	<0.01	27.9	
	2019	134	1.68~25.3	0.05	9.78	0.90	<0.01	372	
	2020	132	0.78~16.9	6.42	3.64	0.93	<0.01	344	
	2021	116	2.03~43.5	4.40	1.83	0.53	<0.01	303	
	2022	52	0.22~19.5	5.00	6.39	0.90	<0.01	292	
	Total	566	0.02~43.5	4.26	5.11	0.84	<0.01		

Note: *W*, the result of the rank-sum (Wilcoxon) test for comparing annual trichloromethane concentrations across years; *p* indicates the *p*-value associated with the W-test. A *p*-value less than 0.05 (*p* < 0.05) indicates a statistically significant difference in trichloromethane concentrations between the years.

**Table 3:** Quarterly trichloromethane concentrations and rank-sum test results.

Indicator	Quarter	Sample Size	Range (μg/L)	Median (μg/L)	Interquartile Range	*W*	*p*	Mean Rank	H (K)
TCM	1	243	0.02~8.83	3.53	3.42	0.96	<0.01	216	94.1
	2	70	0.32~19.5	3.76	2.72	0.78	<0.01	256	
	3	162	0.02~25.3	7.99	9.13	0.95	<0.01	363	
	4	91	1.69~43.5	5.91	9.88	0.77	<0.01	344	

**Table 4:** Detection results for pH, chloride, and turbidity.

Item	Sample Size	Minimum	Maximum	Interquartile Range	Median
pH	566	6.81	8.36	0.25	7.67
Chloride (mg/L)	566	1.00	41.00	9.90	28.60
Turbidity (NTU)	566	0.05	0.64	0.09	0.19

**Table 5:** Exposure factors for calculate health risk of TCM.

Population		IRW (L/d)	BW (kg)	SA (cm^2^)	EF (Day/Year)	ED (Years)	Shower Time (min/Day)	IRa (m^3^/d)
Gender	Female	2.1	57.8	15,000	350	77.4	8	17.7
	Male	2.5	66.1	17,000	350	72.4	8	14.5
Region	Urban	2.4	63.4	16,000	350	80	9	16.3
	Rural	2.3	60.8	16,000	350	70	7	16
	[18, 44)	2.3	61.9	16,000	350	44	9	16.7
Age	[45, 59)	2.3	63.5	16,000	350	59	8	16.7
	[60, 79)	2.2	60.3	16,000	350	79	7	13.8
	80+	1.9	55.5	16,000	350	80	6	12

**Table 6:** Health risk of DBPs in different subgroups.

Variables		HQ_Oral	HQ_Der	HQ_Inh	HI-Total	Risk_Oral	Risk_Der	Risk_Inh	Risk-Total
Gender	Female	1.48 × 10^−2^	1.50 × 10^−3^	3.21 × 10^−4^	1.67 × 10^−2^	5.09 × 10^−6^	5.13 × 10^−7^	1.59 × 10^−8^	5.62 × 10^−6^
	Male	1.54 × 10^−2^	1.48 × 10^−3^	2.30 × 10^−4^	1.72 × 10^−2^	4.95 × 10^−6^	4.76 × 10^−7^	1.07 × 10^−8^	5.44 × 10^−6^
Region	Urban	1.55 × 10^−2^	1.46 × 10^−3^	2.69 × 10^−4^	1.72 × 10^−2^	5.48 × 10^−6^	5.16 × 10^−7^	1.38 × 10^−8^	6.01 × 10^−6^
	Rural	1.55 × 10^−2^	1.52 × 10^−3^	2.76 × 10^−4^	1.72 × 10^−2^	4.79 × 10^−6^	4.70 × 10^−7^	1.24 × 10^−8^	5.27 × 10^−6^
	[18, 44)	1.52 × 10^−2^	1.49 × 10^−3^	2.83 × 10^−4^	1.70 × 10^−2^	2.96 × 10^−6^	2.90 × 10^−7^	7.97 × 10^−9^	3.26 × 10^−6^
Age	[45, 59)	1.48 × 10^−2^	1.45 × 10^−3^	2.75 × 10^−4^	1.65 × 10^−2^	3.87 × 10^−6^	3.80 × 10^−7^	1.04 × 10^−8^	4.26 × 10^−6^
	[60, 79)	1.49 × 10^−2^	1.53 × 10^−3^	2.40 × 10^−4^	1.67 × 10^−2^	5.21 × 10^−6^	5.35 × 10^−7^	1.21 × 10^−8^	5.76 × 10^−6^
	80+	1.40 × 10^−2^	1.66 × 10^−3^	2.26 × 10^−4^	1.59 × 10^−2^	4.95 × 10^−6^	5.89 × 10^−7^	1.16 × 10^−8^	5.56 × 10^−6^

## Data Availability

The data are available from the corresponding author upon reasonable request.
